# Maryland State Veterinary Medical Association—Special Meeting

**Published:** 1892-03

**Authors:** H. A. Meisner

**Affiliations:** Secretary


*Maryland State Veterinary Medical Association.*—A special meeting of the Maryland State Veterinary Medical Association, was held February 5th, at its rooms, corner Madison avenue and Orchard street, with Dr. George C. Faville, President *pro tem*, in chair. There were present Drs. Geo. C. Faville, Wm. Dougherty, T. F. Barron, A. W. Clements, D. R. Hoffman, and H. A. Meisner. The minutes of the previous meeting were read and approved.

The Ambulance Committee reported that they had conferred with Mr. Durall, Secretary of the Society for the Prevention of Cruelty to Animals, who informed them that an ambulance and harness could be procured for five hundred dollars, also that he thought arrangements could be made with the Adams Express Company for furnishing horses, etc.

Under new business, Dr. Wm. Dougherty moved that our Secretary report the meetings of this Association in all the daily papers ; carried.

The proposal of new members being next in order Dr. Hoffman proposed Dr. G. Allen Johnuan, of Chestertown, Md., whose name was referred to the Board of Censors to be reported upon at next meeting. Dr. Dougherty delivered an excellent address upon a new method of treating chronic shoulder and hip lameness, citing some four or five cases, three of which had been successfully treated. Adjourned.

H. A. Meisner, V. M. D., *Secretary.*

